# Perceptions and Experiences of Supplemental Nutrition Assistance Program (SNAP) Participants Related to Receiving Food and Nutrition-Related Text Messages Sent Agency-Wide: Findings from Focus Groups in San Diego County, California

**DOI:** 10.3390/nu15122684

**Published:** 2023-06-09

**Authors:** Celeste Felix, Ron Strochlic, Blanca Melendrez, Hao Tang, Shana Wright, Wendi Gosliner

**Affiliations:** 1 Nutrition Policy Institute, Division of Agriculture and Natural Resources, University of California, Oakland, CA 94607, USA; cmfelix@ucanr.edu (C.F.); rstrochlic@ucanr.edu (R.S.); 2 Center for Community Health, Altman Clinical and Translational Research Institute, University of California San Diego, La Jolla, CA 92037, USA; bmelendrez@health.ucsd.edu (B.M.); smwright@health.ucsd.edu (S.W.); 3 Health and Behavior Studies, Teachers College, Columbia University, New York, NY 10027, USA; ht2489@tc.columbia.edu

**Keywords:** SNAP, nutrition education, mHealth, text messaging, safety net program experiences

## Abstract

We developed and sent a series of five monthly text messages promoting fruit and vegetable consumption to approximately 170,000 SNAP participants in San Diego County, California. The text messages, which were sent in English and Spanish, included links to a dedicated bilingual website offering additional information, including how to select, store, and prepare seasonal fruits and vegetables, health benefits of different fruits and vegetables, recipes, and tips to reduce food waste. To our knowledge, this represents the first instance of a SNAP agency providing nutrition information directly to SNAP participants. We conducted seven focus groups (four in English and three in Spanish) with a convenience sample of twenty-six text message recipients, to elicit their perceptions of this intervention, self-reported behavior changes, and recommendations for moving forward. Respondents reported overwhelmingly positive perceptions of this effort, including increased intake of fruits and vegetables, and trying new fruits and vegetables. Participants also reported improved perceptions of SNAP. Virtually all would like this effort to continue, and many would like to receive the messages more frequently than once a month. This effort represents a relatively low-cost approach that SNAP agencies can implement to provide SNAP participants with food and nutrition information that can help them to improve their diets, optimize their food dollars, and enhance their feelings of well-being related to participating in the program.

## 1. Introduction

Diet-related chronic disease is a significant public health concern in the US. Nearly one in ten Americans have been diagnosed with diabetes [1] and about half of American adults suffer from cardiovascular disease [2]. An improved diet, including increased intake of fruits and vegetables (FV), can help reduce the risk of diet-related chronic disease [3]. However, only 1 in 10 adults eat the recommended amounts of FV [4]. 

The Supplemental Nutrition Assistance Program (SNAP) is the nation’s largest federal nutrition assistance program, with over 41 million Americans participating in an average month in 2021 [5]. Despite providing financial resources to support food purchases, SNAP does not typically provide participants with education promoting or facilitating a healthful diet. The Supplemental Nutrition Assistance Program Education (SNAP-Ed) provides evidence-based interventions, including education, to promote a healthy diet and physical activity. However, SNAP-Ed’s scope is small, and most resources are directed at critical public health interventions that change the policies, systems, and environments that shape participants’ food experiences and food choices. In 2018, SNAP-Ed provided direct education to 3.8 million people, representing less than 10% of SNAP participants [6].

Use of mobile health interventions, including text messaging and websites to promote the adoption of more healthful behaviors, has been increasing in recent years [7,8,9]. Systematic reviews have shown mixed results of these interventions [10,11]. Evaluations of specific interventions have shown mixed results as well, with some reporting positive effects [12,13,14] while others did not [15,16,17]. 

Considering the broad reach and relatively low cost of mobile health interventions, we implemented and evaluated a pilot intervention sending text messages from a SNAP agency to SNAP participants, promoting FV intake, with links to a dedicated website (eat-ca.org) developed by the study team offering more detailed information about how to select, store, and prepare local, seasonal fruits and vegetables. We partnered with the County of San Diego Health and Human Services Agency (HHSA), the SNAP agency in San Diego County, California, which was already utilizing an opt-out text message system to provide administrative reminders and alerts to all CalFresh participants throughout the county. Findings from a survey of program participants [18] indicated significant increases in knowing where to obtain information about selecting, storing, and preparing FV, feeling good about participating in SNAP, and thinking the SNAP program helps participants eat healthfully. No significant pre/post-differences were found in fruit or vegetable consumption, measured using two modified self-reported intake questions from the university of California Food Behavior Checklist [19], although 68% of participants self-reported increased FV intake at the follow-up. Nearly all respondents appreciated the intervention and wanted it to continue. This paper presents findings from focus groups we conducted to better understand participant perceptions of the program and recommendations for improvements. 

## 2. Materials and Methods

### 2.1. Intervention 

All San Diego County SNAP participants opting into the County’s text messaging system received monthly text messages in English or Spanish between September 2020 and February 2021. The messages promoted the consumption of California-grown fruits and vegetables and included links to a website with further information, including how to select, store, and prepare FV, recipes, and tips for reducing food waste. The design of the text messages followed the behavior change technique taxonomy, which consists of 93 behavior change techniques arranged in a hierarchical order. The specific techniques used included providing pros and cons and persuasive argumentation to explain the benefits of fruit and vegetable intake, highlighting cost-savings associated with seasonal and local produce to shape knowledge, offering actionable messages and links to a credible website to facilitate action planning, and promoting a sense of altruism by supporting local growers, thereby creating a “warm glow” associated with benefiting others. A prior publication provides a detailed description of the text messages and website development [18]. 

### 2.2. Recruitment 

**Figure 1:**
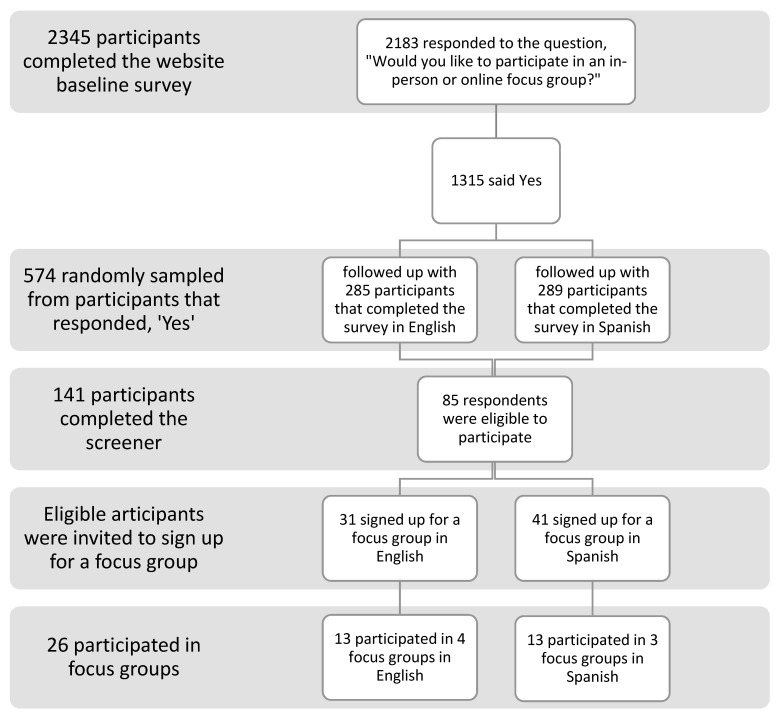
Flow chart of recruitment process.

We posted an invitation to participate in an online survey on the www.eat-ca.org website after sending the first text message. The survey elicited interest in participating in a focus group. Participants were told they would receive a USD 50 gift card as thanks for participation. Of the 2345 respondents that completed the survey, 1315 (56.1%) expressed interest in participating in a focus group and provided their contact information. We sent text messages inviting participation in a focus group in batches of up to 50 participants at a time, equally divided between English and Spanish speakers. The focus group invitation included several screener questions. Participants were required to be at least 18 years old, be current or recent SNAP participants, recalled receiving the intervention text messages, visited the website at least once, and had access to Zoom, the platform used to conduct the online focus groups. Ultimately, 574 individuals were invited to participate in focus groups. Of those, 141 (24.6%) completed the screener (68 English, 73 Spanish). Fifty-six respondents answered “no” or “not sure” to at least one screener question and were excluded from the sample. The 85 respondents eligible to participate in the focus groups were asked to sign up for one of two different time slots during the day and evening. Daytimes ranged from 12 to 2 p.m. and evening times were at 5 pm. Seventy-two people signed up for focus groups. We sent text message reminders and calls the day of the focus groups and 26 people attended. Participants received a USD 50 gift card as a thank you for their involvement. The recruitment process is depicted in Figure 1.

### 2.3. Focus Groups

The focus group guide was designed to gain insights into participants’ perceptions of the monthly text messages and the accompanying website (Table 1). Three focus groups were initially conducted in English and three in Spanish. However, due to low turnout in the English focus groups, an additional English language focus group was conducted. Two experienced members of the research team were present at each focus group, with one facilitating and the other taking notes. All focus group discussions were held on Zoom and recorded with participants’ permission. The discussions lasted between 45 and 60 min. 

### 2.4. Analysis

Zoom automatically generated preliminary English transcripts. A member of the research team reviewed each transcript against the recording to rectify any errors. An outside firm transcribed the Spanish recordings, and a professional translator translated the Spanish transcript. Once all transcripts were cleaned, two researchers, who were also note-takers for the focus groups, immersed themselves in the data by reading through each transcript. Through the immersion process, clear themes emerged, and a codebook was drafted. Carefully coding each transcript using an inductive coding approach, one researcher added codes to the codebook. After all the transcripts were coded and the codebook was finalized, the rest of the study team double-checked the codes and created memos throughout the transcripts that helped summarize the key findings. 

## 3. Results

A total of 26 participants attended the focus groups (13 English, 13 Spanish). Participants in focus groups conducted in Spanish were almost exclusively female (92%) and all but one participant identified as Latino/a (92%). In contrast, 69% of participants in the English language focus groups were female, and those groups were more racially/ethnically diverse (Table 2). 

**Figure 2:**
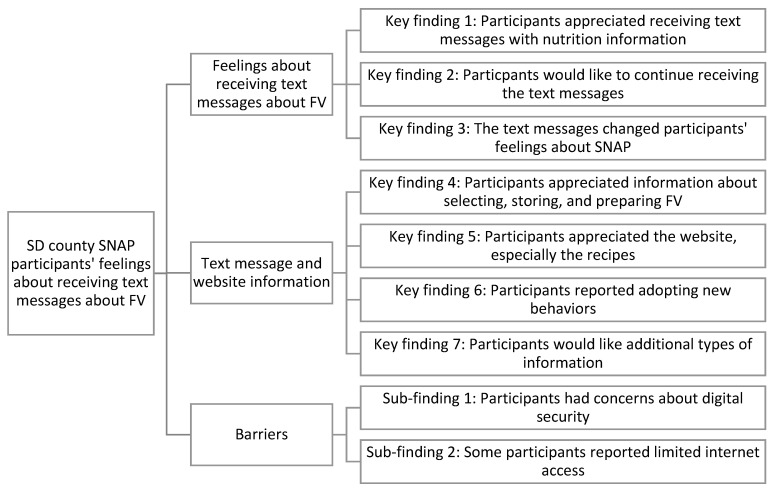
Findings that emerged from focus groups with SNAP participants in San Diego County.

We identified seven key findings and two sub-findings based on the focus group discussions (Figure 2). Key findings mentioned in almost every focus group were that participants appreciated receiving the text messages with information about fruits and vegetables and that all participants expressed a desire to continue receiving the text messages. Some reported improved perceptions of the SNAP program, noting that the text messages made them feel that the SNAP agency cared about their health and well-being. 

Participants also appreciated the information in the text messages about selecting, storing, and preparing CA-grown fruits and vegetables, as well as the health and nutrition facts about the highlighted produce items. They also found the dedicated website providing more in-depth information and recipes to be useful. Participants reported trying recommended fruits for the first time and preparing recipes from the website. Participants offered numerous recommendations for additional information they would like to receive in the future, including advice for people with diabetes, tips for feeding picky-eaters, and how to eat to strengthen one’s immune system. A sub-finding from the discussions was that some participants were unable to access the website due to limited internet access. Additionally, some expressed initial concerns about clicking on the text message links due to fears of viruses or scams (Table 3).

## 4. Discussion

Participants in our study reported making behavioral changes, including increasing their FV intake and trying new varieties, changing how they select, store, and prepare FV, and shopping at farmers’ markets. Participants also reported greatly appreciating receiving these messages. They valued information about the health benefits of different FV, awareness that seasonal fruits and vegetables can be more affordable, and money-saving tips for reducing food waste. Participants also appreciated the website, particularly the recipes highlighting the featured FV. All participants expressed a desire to continue receiving the messages and most would like to receive them more frequently. A focus group participant no longer receiving SNAP expressed a desire to continue receiving the messages, despite no longer receiving SNAP benefits. The messages also had a positive impact on participants’ perceptions of SNAP. Many expressed appreciation for SNAP’s concern for their health and some felt grateful that they no longer felt “like a number.” The messages were sent during the COVID-19 pandemic and some participants also reported that they made them feel less alone. 

The SNAP program plays a vital role in helping people with low incomes access food. It has been associated with improved household food security [20], improved health outcomes for adults that received SNAP benefits as children, and improved economic outcomes for women [21]. The SNAP program routinely sends administrative messages to ensure that eligible participants maintain their benefits; however, despite providing over USD 113 million in food-related benefits to over 41 million people in the US [22], there is no established mechanism for providing SNAP participants with information that can help them make healthful food choices. The SNAP-Ed program promotes improved diet and nutrition among all SNAP-eligible community members; however it does not target SNAP participants directly and reaches a very limited number of them. 

The San Diego County SNAP agency routinely sends text messages with administrative messages to all SNAP participants in the county. Studies have found that mobile health approaches, including text messages and websites, can be an effective and acceptable way of disseminating nutrition and other health-related information in general [7,8]. Some prior studies of text-messaging interventions found them to be effective at increasing fruit and vegetable intake [12,13], while others showed no or mixed results [15,16,17]. Our pilot intervention consisted of sending five monthly text messages with information about the health and other benefits of local and seasonal fruits and vegetables, with links to a dedicated website providing more in-depth information, recipes, and links to additional resources. To our knowledge, this is the first instance of a SNAP agency sending text messages of this nature to its entire caseload (except those who had opted out). 

Future opportunities exist to address challenges and incorporate recommendations provided by participants. Challenges included limited internet access, which made access to the website challenging for some. Some also expressed initial concerns that the messages might be “spam” containing viruses. Conversely, many participants expressed confidence in the messages since they came from UC San Diego, considered a trusted source. Focus group participants also noted limited awareness of the links to additional resources on the website, indicating a need to make those more apparent. 

Participants offered recommendations for additional information they would like, including diets for diabetics, heart-healthy diets, reducing inflammation, and feeding picky children. Funding for this pilot limited the information we could provide to the promotion of California-grown FV. However, the San Diego SNAP agency has continued to send the text messages beyond the pilot period and has requested additional messages. Future efforts could also incorporate these types of additional messaging for recipients.

This study adds to the relatively scant literature on the use of behavioral science to inform mobile health interventions [23], particularly in a large public program such as SNAP. This effort is relatively easy for other SNAP agencies with text messaging capacity to replicate, and future studies should more rigorously test the effects of this intervention. Some challenges to implementation remain, however. Sending the messages has required approximately 5 h of staff time each month to send 170,000 messages at a cost of USD 0.02 per message. The San Diego SNAP agency has utilized an opt-out approach, according to which SNAP participants receive messages unless they specifically request not to. Agencies using an opt-in approach to text messages may need to invest greater resources in order to recruit participants.

### Limitations

This study has several limitations. The focus group participants were recruited based on interest in participating in a focus group via a survey administered to text message recipients that had also visited the study website, likely creating selection bias. They represent a more engaged subset of text message recipients and as such, the findings may not be generalizable to the wider SNAP population. The focus groups were conducted online using Zoom, limiting the collection of demographic information, and limiting participation to those with access to that technology. This may have excluded individuals who do not have access to reliable internet or who may not be comfortable with virtual communication methods, which could have affected the diversity of participants and their perspectives. Additionally, only 5 of the 26 focus group participants were men, making it difficult to generalize our findings to other male SNAP participants. Furthermore, while the behavior change techniques used in the text messages were based on a well-established taxonomy, the study did not individually assess the effectiveness of each technique. Lastly, the study was conducted over a relatively short period of six months, and as such, it does not reflect longer-term effects or consider the sustainability of the program.

Overall, this study provides valuable insights into the experiences and preferences of a subset of SNAP participants exposed to an innovative text message program administered by their SNAP agency. Further research is needed to assess the effectiveness of the intervention using a more rigorous study design, in a larger and more diverse population, and over a longer period of time. Future studies should also examine long-term health impacts and behavior change, as well as household composition and impacts on other family members.

## 5. Conclusions

The results of this study suggest that the use of short text messages focused on food and nutrition can be a valuable tool for improving the health and well-being of SNAP participants. With as little as 200 characters, the intervention was successful in encouraging participants to try new foods and recipes and engendered more positive feelings toward the SNAP program. These findings provide a promising basis for future research into the effectiveness of this type of intervention in SNAP agencies. More rigorous testing is needed to further evaluate the impact of SNAP agency-wide text message interventions on SNAP participants’ nutrition and health outcomes. If successful, this approach could be scaled up to additional local and statewide SNAP agencies, potentially providing meaningful benefits to more SNAP participants.

## Figures and Tables

**Table 1:** Focus group questions asked of SNAP participants participating in the text intervention in San Diego County.

Focus Group Guide
1. We’re interested in your thoughts on the text messages about fruits and vegetables that you got from CalFresh. How did you feel about getting these text messages? ∙ Probe: Did they feel different from the usual messages you get from CalFresh? What words or feelings come to mind?
2. How did getting those messages make you feel about CalFresh? Was that any different from how you felt before?
3. I’m going to share my screen and show you some of the text messages to refresh your memory [SHARE SCREEN AND DISPLAY MESSAGES]. What other thoughts come up for you on seeing these messages? What suggestions do you have for improving the text messages? Are there other ways you would prefer to get this information? ∙ Probe: content, FV highlighted, length, frequency, delivery (text vs. email), other
4. We’re also interested in your thoughts about the website that the text messages linked to. What do you remember about the website? What did you like about it? What didn’t you like?
5. What other thoughts or feelings come up for you on seeing the website? What do you think of the different sections? What suggestions do you have for improving the website? ∙ Probe: content, visuals, organization, navigation, interactivity/engagingness
6. The text messages and website provided information about how to select, store, and prepare different fruits and vegetables, with recipes and tips for reducing food waste. Which aspects did you find most useful? What additional information would you like?
7. Thinking about the text messages and website, what information was new or especially helpful for you?
8. Can you tell us about any changes you’ve made in selecting, storing, or preparing fruits and vegetables, wasting food, or buying local or seasonal produce because of the text messages or website?
9. CalFresh has been sending these messages for about a year. How would you feel about continuing to get these types of messages? ∙ Probe: How would you feel if you stopped getting them?
10. All the messages so far have been about fruits and vegetables. What additional information would you like to get by text from CalFresh?
11. Those are all the questions I have for you. Is there anything else you’d like to share about the text messages and website?

**Table 2:** Description and demographic characteristics of each focus group conducted with SNAP participants in SD County.

Focus Group #	Month	Time of Day	# of Participants, *n* = 26	Language	Gender	Race/Ethnicity
1	November 2021	Evening	2	Spanish	2 Female	2 Latino/Latina
2	November 2021	Midday	4	Spanish	4 Female	3 Latino/Latina 1 White
3	December 2021	Evening	7	Spanish	6 Female 1 Male	7 Latino/Latina
4	November 2021	Evening	2	English	2 Female	1 White/Latina 1 White
5	November 2021	Midday	3	English	1 Female 2 Male	1 Asian or Asian American 1 White 1 Latino/Latina
6	December 2021	Evening	5	English	3 Female 2 Male	1 Asian or Asian American 1 Black or African American 1 White 1 Latino/Latina 1 Refused to answer
7	December 2021	Evening	3	English	3 Female	1 Black or African American 2 White

**Table 3:** Findings (in bold type) and supporting quotations (in italics) from focus groups with SNAP participants in San Diego County.

**Feelings about Receiving Text Messages about FV**
**Key Finding 1: Participants greatly appreciated receiving text messages with nutrition information**
1.1 *I just really appreciated it, I think it’s really amazing. It’s like something that you sign up for, you know, when you sign up for those things that you see, like on Facebook or something… and they get you these, you know, foods that you should try and recipes that you should try and, you know, let you know what to eat and how much and you know, I just think it’s really important and I’m grateful for it.*
-White Female (English)
1.2 *… it does remind me to think of it as a way to eat healthier. It’s kind of like when I get the messages, it’s like, oh, yeah, you know, I get a little bit extra. And that enables me to buy healthier foods. So, it is a good little reminder.*
-White Female (English)
1.3 *Thank you very much for helping us…making us feel that…we can find options, know more, learn more. Because sometimes you forget that you need to keep up with information.*
-Latina Female (Spanish)
**Key Finding 2: Participants expressed they would like text messages to continue and many would prefer to receive them more frequently**
2.1 *Oh, well, yes, of course I would like to keep getting them. Well, more often would be good, too, because that way… you learn more, instead of being forgotten for a month.*
-Latina Female (Spanish)
2.2 *So I would be really bummed [if the messages stopped] because for me the information is really valuable.*
-White Female (English)
2.3 *I really liked the text messages. I think for a month I did not get them, and I was like, awww [making sad face] because I really liked the nutritional facts.*
-Asian Female (English)
2.4 *For these, I wouldn’t have minded getting them like once a week, that would have been fine with me.*
-Latina Female (Spanish)
**Key Finding 3: Participants explained that the text messages about FV changed their feelings about SNAP, making them feel cared about and connected**
3.1 *It’s more human… it implies that there’s some care about the person behind the…institution. When you’re in the welfare system in any way, even if the only part you’re getting is food stamps—I’ve gotten the full gamut throughout my life at different periods. It’s very dehumanizing. And you often feel a lot of negative emotions or feelings about it, embarrassment, shame, and just frustration that you’re just a number and things like that. I guess there’s a sense that when you get a text message, that’s cheerful and yeah, it’s information, there’s a little bit of a feeling that it’s more than that, that it’s caring about your health and your well-being. And I guess even though I know that it’s kind of coming from an institution also, it just has a different feeling about it for me. I think it’s, it shows that things are moving maybe in a more positive direction when it comes to public programs.*
-White Female (English)
3.2 *I felt important. I felt that I was important to somebody, my, my eating habits, right? And, basically for my son’s eating habits. It feels good to have someone close by, helping you with tips.*
-Latina Female (Spanish)
3.3 *Oh my goodness. It just made me feel like, they care so much. You know, it was like, it made it personal for me… So, like, more intimate for me to have a relationship with, with CalFresh.*
-Latina Female (English)
Feelings about the information
**Key Finding 4: Participants expressed their appreciation for information regarding selecting, storing, and preparing FV**
4.1 *Okay, it tells us in the link, it tells us how we can, um, select green beans. Choose fresh green beans that are thin. A lot of times we don’t know about the, um, vegetables, we don’t know which ones are the best that are in the best condition, or that are tender, that have the best texture for buying and, um, eating. So this tells us how they should look, including bright green color… Then how we should store them. I would like you to keep telling us about all this because sometimes we don’t know.*
-Latina Female (Spanish)
4.2 *I thought it was very interesting… for example, eat what’s in season because it’s cheaper, because they have more vitamins… it made me look for more information, so… it piqued my curiosity.*
-Latina Female (Spanish)
4.3 *… that’s one of my favorite parts because you, they give you tips, like I said before, on how to prepare a food or something you have at home. And well, obviously, well, you adjust it, whether to your budget or to your preferences, well, in this case, my kids are what I struggle with the most, to get them to eat fruits and vegetables. I adjust it to what they like, too, and I try to make changes. For me, that part is perfect. I loved it.*
-Latina Female (Spanish)
4.4 *Well, it’s knowing the quality of each fruit, what it contributes, to health, especially what time of year can you buy it, because that also helps you save. And yes, I have known that when there’s fruit in season, you need to buy it and use it and eat it because it really is cheaper than when it’s not in season, so I’m very interested in all this information, and I follow it and try to do it.*
-Latina Female (Spanish)
**Key Finding 5: Participants expressed their appreciation for the website, especially because of the recipes**
5.1 *For me, it was the recipes for the most part, you know, looking at different, different ideas that maybe I hadn’t thought of… that part was very helpful.*
-White Male (English)
5.2 *I’ve even taken recipes to places when I’m, you know, going to maybe like a small gathering of friends. I’ll take a recipe. I took a recipe of vegetable and rice that you guys had given out. And I took that with me and my friend said, ’Oh, honey, this is delicious.’ I was like I got it from food stamps.*
-White Female (English)
5.3 *One of the essential tools that they tell us and that by nature is, besides what the doctor tells us, is eating food in a healthier way. Like for example, boiled green beans instead of fried, put them in the blender when they’re warm just boiled, eat them in soup. All those kinds of situations are going to help us to at least be a little healthier so we can counteract disease, and at the same time feel good, actually with ourselves and our family. All the products you’ve given us and the, and the recipes that you’ve also given us, I’ve made them here. The last time I made a recipe that I liked, that was a salad of all vegetables.*
-Latino Male (Spanish)
**Key Finding 6: Participants reported adopting new behaviors related to FV purchase, storage, preparation, and consumption as a result of receiving the messages and/or using the website**
6.1 *Like, I look up, and now I go to a farmers’ market. And I try to buy local now, I never, never even thought about that. I hate to say that, but it was something I never thought about until you guys sent me that.*
-White Female (English)
6.2 *Can I tell you, I had never had persimmons. So after I read all the nutritional value of persimmons, I went and got, like three. I’ve never had one…They’re delicious… I had persimmons and kiwi and…I am eating bell peppers now. I never really ate bell peppers.*
-White Female (English)
6.3 *I received the message about persimmons. I already knew about them. I had never tasted them, but thanks to the link…we went and bought some and loved them.*
-Latina Female (Spanish)
6.4 *I do remember getting the kiwi one as well. And I’m not a real big kiwi fan, but I did go out and buy some and, and try them out. And I mix those in smoothies as well, which I thought was quite interesting.*
-Latino Male (English)
6.5 *Well, I felt very privileged to get those because every time there was a text message, and there was a fruit suggested that we try with the…details and all that. If I wasn’t trying it already, I went out and I bought it and tried it. And now it’s a part of my regular diet.*
-White Female (English)
**Key Finding 7: Participants would like to receive many other types of information**
7.1 *I like how you’re talking about the composting and about that, like, if we get a text message about that, maybe it’ll encourage more people to do it, like, see it not waste as much food. Those are good ideas too.*
-Asian Female (English)
7.2 *I still would like to know something about drinks. You know, I’m trying to move away from soda completely to incorporate more water, but it just we be missing alternatives to just water, healthy drinks. Not sugary, sugar laced, you know, calorie concentrated. Like what, I don’t, yeah, you don’t know what else to drink besides water.*
-Didn’t wish to answer Female (English)
7.3 *Yeah. Anti-inflammatory, anti-sugar. You know, I mean, I mean, I know that’s really asking a lot that you know, just once in a while like to get a sugar-free, flour-free sweet.*
-White Female (English)
Barriers
**Sub-Finding 1: Participants expressed concerns about clicking on the link, but noted that the SNAP agency is a trusted source**
8.1 *At first, my response was to not pay too much attention, but um, since I saw it came from, from CalFresh, right? From the county, then I felt sure, sure it was real because, well, we’ve been warned not to fall for the trap of people who aren’t who they say they are.*
-Latina Female (Spanish)
8.2 *Nowadays it’s mainly, because of the situation, whether it’s phone calls, if it’s fraud scams. So, I would think along those lines about not trusting. Or you click on it and…maybe it already sent you to another link, or they want to steal your information. I would think along those lines about not trusting, thinking it might be spam or something like that, or someone wants to go in and steal your information from the beginning.*
-Latina Female (Spanish)
**Sub-Finding 2: Participants explained that limited internet access was a barrier to visiting the website**
9.1 *Supposedly I have unlimited Internet, supposedly. I pay, but it’s not true. You use like five gigs and there’s no more. So the, the five few gigabytes I have for the whole month, I try to save them however I can, right? Not watching or looking at too many things that use up my Internet if I’m away from home.*
-Latina Female (Spanish)
9.2 *The truth is, I like texts better because I’m more, like, I don’t have much Internet on the phone, and text messages, I can get them. So I imagine that, like me, there’s a lot of people, right?*
-Latina Female (Spanish)

## Data Availability

The data are available from the corresponding author upon reasonable request.

## References

[1] Centers for Disease Control and Prevention National Diabetes Statistics Report. https://www.cdc.gov/diabetes/data/statistics-report/index.html.

[2] American Heart Association News Cardiovascular Diseases Affect Nearly Half of American Adults, Statistics Show. https://www.heart.org/en/news/2019/01/31/cardiovascular-diseases-affect-nearly-half-of-american-adults-statistics-show.

[3] Wallace T.C., Bailey R.L., Blumberg J.B., Burton-Freeman B., Chen C.-Y.O., Crowe-White K.M., Drewnowski A., Hooshmand S., Johnson E., Lewis R. (2020). Fruits, vegetables, and health: A comprehensive narrative, umbrella review of the science and recommendations for enhanced public policy to improve intake. Crit. Rev. Food Sci. Nutr..

[4] Centers for Disease Control and Prevention Only 1 in 10 Adults Get Enough Fruits or Vegetables. https://www.cdc.gov/nccdphp/dnpao/division-information/media-tools/adults-fruits-vegetables.html.

[5] Jones J.W., Toossi S., Hodges L. (2022). The Food and Nutrition Assistance Landscape: Fiscal Year 2021 Annual Report.

[6] Puma J.E., Young M., Foerster S., Keller K., Bruno P., Franck K., Naja-Riese A. (2021). The SNAP-Ed evaluation framework: Nationwide uptake and implications for nutrition education practice, policy, and research. J. Nutr. Educ. Behav..

[7] Duan Y., Shang B., Liang W., Du G., Yang M., Rhodes R.E. (2021). Effects of eHealth-based multiple health behavior change interventions on physical activity, healthy diet, and weight in people with noncommunicable diseases: Systematic review and meta-analysis. J. Med. Internet Res..

[8] Sahin C., Courtney K.L., Naylor P.J., E Rhodes R. (2019). Tailored mobile text messaging interventions targeting type 2 diabetes self-management: A systematic review and a meta-analysis. Digit. Health.

[9] Head K.J., Noar S.M., Iannarino N.T., Harrington N.G. (2013). Efficacy of text messaging-based interventions for health promotion: A meta-analysis. Soc. Sci. Med..

[10] Müller A.M., Alley S., Schoeppe S., Vandelanotte C. (2016). The effectiveness of e-& mHealth interventions to promote physical activity and healthy diets in developing countries: A systematic review. Int. J. Behav. Nutr. Phys. Act..

[11] Marcolino M.S., Oliveira J.A.Q., D’Agostino M., Ribeiro A.L., Alkmim M.B.M., Novillo-Ortiz D. (2018). The impact of mHealth interventions: Systematic review of systematic reviews. JMIR Mhealth Uhealth.

[12] Gustafson A., Jilcott Pitts S.B., McQuerry K., Babtunde O., Mullins J. (2019). A Mentor-Led Text-Messaging Intervention Increases Intake of Fruits and Vegetables and Goal Setting for Healthier Dietary Consumption among Rural Adolescents in Kentucky and North Carolina, 2017. Nutrients.

[13] Pedersen S., Grønhøj A., Thøgersen J. (2016). Texting your way to healthier eating? Effects of participating in a feedback intervention using text messaging on adolescents’ fruit and vegetable intake. Health Educ. Res..

[14] Santo K., Hyun K., de Keizer L., Thiagalingam A., Hillis G.S., Chalmers J., Redfern J., Chow C.K. (2018). The effects of a lifestyle-focused text-messaging intervention on adherence to dietary guideline recommendations in patients with coronary heart disease: An analysis of the TEXT ME study. Int. J. Behav. Nutr. Phys. Act..

[15] Islam S.M.S., George E.S., Maddison R. (2021). Effectiveness of a mobile phone text messaging intervention on dietary behaviour in patients with type 2 diabetes: A post-hoc analysis of a randomised controlled trial. Mhealth.

[16] Power J.M., Bersamin A. (2018). A Text Messaging Intervention (Txt4HappyKids) to Promote Fruit and Vegetable Intake Among Families With Young Children: Pilot Study. JMIR Form. Res..

[17] Dobson R., Whittaker R., Jiang Y., Maddison R., Shepherd M., McNamara C., Cutfield R., Khanolkar M., Murphy R. (2018). Effectiveness of text message based, diabetes self management support programme (SMS4BG): Two arm, parallel randomised controlled trial. BMJ.

[18] Gosliner W.F.C., Strochlic R., Wright S., Yates-Berg A., Thompson H., Tang H., Melendrez B. (2023). Feasibility and Response to the San Diego County, California, Supplemental Nutrition Assistance Program (SNAP) Agency Sending Food and Nutrition Text Messages to All Participants: Quasi-Experimental Web-Based Survey Pilot Study. J. Med. Internet Res..

[19] Blackburn M., Townsend M., Kaiser L., Martin A., West E., Turner B., Joy A. (2006). Food behavior checklist effectively evaluates nutrition education. Calif. Agric..

[20] Mabli J. (2015). Supplemental Nutrition Assistance Program participation is associated with an increase in household food security in a national evaluation. J. Nutr..

[21] Hoynes H., Schanzenbach D.W., Almond D. (2016). Long-Run Impacts of Childhood Access to the Safety Net. Am. Econ. Rev..

[22] United States Department of Agriculture, Food and Nutrition Service SNAP Data Tables—National and/or State Level Monthly and/or Annual Data. https://www.fns.usda.gov/sites/default/files/resource-files/34SNAPmonthly-12.pdf.

[23] Voorheis P., Zhao A., Kuluski K., Pham Q., Scott T., Sztur P., Khanna N., Ibrahim M., Petch J. (2022). Integrating Behavioral Science and Design Thinking to Develop Mobile Health Interventions: Systematic Scoping Review. JMIR mHealth uHealth.

